# Accumulation of Aluminium and Physiological Status of Tree Foliage in the Vicinity of a Large Aluminium Smelter

**DOI:** 10.1100/2012/865927

**Published:** 2012-05-02

**Authors:** E. D. Wannaz, J. H. Rodriguez, T. Wolfsberger, H. A. Carreras, M. L. Pignata, A. Fangmeier, J. Franzaring

**Affiliations:** ^1^Section of Pollution and Bioindicators, Multidisciplinary Institute of Plant Biology, Faculty of Physical and Natural Sciences, National University of Córdoba, Avenida Vélez Sársfield 1611, X5016CGA Córdoba, Argentina; ^2^Institute for Sustainable Waste Management and Technology, University of Leoben, Franz Josef-Straße 18, 8700 Leoben, Austria; ^3^Plant Ecology and Ecotoxicology Section, Institute of Landscape and Plant Ecology (320), University of Hohenheim, August-von-Hartmann-Straße 3, 70599 Stuttgart, Germany

## Abstract

A pollution gradient was observed in tree foliage sampled in the vicinity of a large aluminium production facility in Patagonia (Argentina). Leaves of *Eucalyptus rostrata, and Populus hybridus* and different needle ages of *Pinus* spec. were collected and concentrations of aluminium (Al) and sulphur (S) as well as physiological parameters (chlorophyll and lipid oxidation products) were analyzed. Al and S concentrations indicate a steep pollution gradient in the study showing a relationship with the physiological parameters in particular membrane lipid oxidation products. The present study confirms that aluminium smelting results in high Al and sulphur deposition in the study area, and therefore further studies should be carried out taking into account potentially adverse effects of these compounds on human and ecosystem health.

## 1. Introduction

Air pollution has been reported to cause extensive damage to the vegetation in the vicinity of aluminium smelters [1–4]. Among the pollutants emitted into the atmosphere by this activity are fluoride compounds, aluminium, polycyclic aromatic hydrocarbons (PAH), polychlorinated dibenzo-p-dioxins, dibenzofurans, and traces of heavy metals [5–7]. Because plant foliage is effectively scavenging atmospheric pollutants, many studies have used different plant species as biomonitors of airborne pollutants in the vicinity of such facilities [1, 4, 7, 8]. Particulate matter (PM) containing the pollutants is deposited on the vegetation surrounding the industry and also enters the soil via rain as water-soluble components, as dust, and contaminated litter [7]. Biomonitoring employing foliage of vegetation is an effective tool for detecting potential health and environmental risks in the vicinity of pollutant emission sources, as has been demonstrated in biomonitoring networks studies [9].

Due to the increasing industrialisation in many developing countries and emerging economies, aluminium industries have increased their production significantly. Aluminium is used extensively in food packaging, construction, and transportation, and associated industries [6]. The Al production comprises two stages, obtaining aluminium oxide by the Bayer process from bauxite and subsequent electrolysis of oxide to obtain elemental aluminium (electrolytic Hall-Héroult process). During this latter process, aluminium particles are emitted among other pollutants (HF, PAH) into the atmosphere. Once emitted into the atmosphere, metallic particles may be respired and deposited in the environment [10]. Several reports showed that aluminium is a very potent neurotoxicant and can disrupt parathyroid hormones [6]. Furthermore, it has been claimed that neurodegenerative diseases like Alzheimer and amyotrophic lateral sclerosis are associated with the Al content in the human brain [11–13]. However, there is little information available on the amount of aluminium particles emitted around Al smelters [3]. Therefore, emission control of pollutants associated with this industry should be made; however, in developing countries these controls are restricted to sporadic investigations and are often insufficient to identify and avoid harmful effects on the environment [14, 15].

One of the largest aluminium production plants of South America is situated on the Atlantic coast of Chubut, Patagonia, Argentina. Some ecological implications of the industrial activity in this area had been described in the early 1980s, when the production capacity was about 140 Kt year^−1^ [16–19]. Although the plant actually has expanded its activities with a yearly production of about 410 Kt [20] contributing approximately 1% of the world production of Al [21], environmental monitoring studies are still scarce. In a recent work we reported high F deposition in biomonitoring performed in the vicinity of the described smelter in Patagonia [22]. Therefore, the objective of this study was to evaluate the environmental impact of the Al smelter on the physiological status and aluminium concentrations in plant foliage growing in the vicinity of the described smelter in Patagonia. Since this area is characterized mainly by tourist activities, the quality of the environment may have implications on human and environmental health.

## 2. Materials and Methods

### 2.1. Study Area and Sampling

The study was performed in the city of Puerto Madryn in the Chubut province (Argentina) in the vicinity of an aluminium production plant. The city is located on the west coast of the Golfo Nuevo and has a population size of about 57,000 inhabitants [23]. However, this number doubles during the summer holidays because the place is one of the most important resorts in Patagonia and the country. The continental climate is characterized by semiarid conditions, with a mean annual precipitation of 238 mm year^−1^ and an average annual temperature of about 13.6°C [24]. The topography of the area is nearly flat, and strong south-westerly winds from the continent to the sea dominate throughout most of the year. Leaves of *Eucalyptus rostrata* and *Populus hybridus* and needles of *Pinus* spec. were collected at forty sampling points in the vicinity of the aluminium factory in the third week of February 2010. Samples consisted of 150–200 leaves or needles and were randomly collected in each sampling site from a single tree according to the standardised method after VDI [25]. During the sampling no precipitation occurred, the precipitation sum from 1st of January 2010 until the date of collection amounted to 24.6 mm and the mean temperature during this time interval was 20.5°C [24]. Leaf area of the samples was determined on an aliquot of 50 leaves using a scanner HP (Scanjet 2200c, Hewlett-Packard, USA) and the software Image J version 1.42q including a ruler in the scanning field as reference.

### 2.2. Physiological Determinations

The procedure followed for the quantification of chlorophyll a (Chl-a), chlorophyll b (Chl-b), phaeophytin a (Phe-a), phaeophytin b (Phe-b), hydroperoxy-conjugated dienes (HPCD), and malondialdehyde (MDA) in leaves of *Eucalyptus rostrata* and *Populus hybridus* and *Pinus* spec. needles consisted of measurements performed in the manner previously described by Carreras et al. [26] and Wannaz and Pignata [27]. Air-dried and milled material was used.

Three subsamples were produced from each sample per sampling point. All concentrations were expressed on a dry weight basis (g^−1^ DW).

### 2.3. Sulphur Concentrations

Five mL of Mg (NO_3_)_2_ saturated aqueous solution was added to 0.3 g plant material and dried in an electric heater. Subsequently, the sample was heated in an oven for 30 min at 500°C. The ashes were then suspended in 6 M HCl and filtered, and the resulting solution was boiled for 3 minutes. Finally the solution was brought to 50 mL with distilled water. The amount of SO_4_^−2^ in the solution was determined by the acidic suspension method with BaCl_2_ which subsequently allowed calculating the sulphur content of each sample [28]. Results were expressed in mg g^−1^ DW.

### 2.4. Aluminium Content in Different Tree Foliage

The concentrations of Al were analyzed in leaves of *Eucalyptus rostrata* and *Populus hybridus* and *Pinus* spec. needles. The plant material (1 g DW) was ground and ashed at 500°C for 4 hours. The ashes were digested with 3.6 mL concentrated HNO_3_, the solid residue was separated by centrifugation, and the volume was adjusted to 25 mL with Milli-Q water. Thereafter the concentration of total Al was determined by graphite furnace atomic absorption spectrometry (THGA Graphite Furnace, Perkin Elmer).

As a quality control, blanks and samples of the standard reference material “CTA-OTL-1” (oriental tobacco leaves, Institute of Nuclear Chemistry and Technology) were prepared in the same way and were run after ten determinations to calibrate the instrument and monitor potential sample contamination during analysis. The results were found to be within 92% and 86%, respectively, of the certified value, with the data indicating a low error of typically less than 15%. The coefficient of variation of replicate analyses was calculated for different determinations. Variations were found to be less than 10%.

### 2.5. Statistical Analysis

Results are expressed as the mean value ± standard deviation (SD) of three determinations for each of the sampling sites. Physiological parameters, aluminium, sulphur, and F concentrations, this last parameter described in Rodriguez et al. [22], were submitted to Pearson's coefficient of correlation in order to study the relationship among the Al and sulphur contents, Al and fluoride content, and physiological variables measured in the foliage of tree species.

Regression analysis was calculated in order to determine relationships between the accumulation of Al, sulphur, and the emission source distance.

## 3. Results and Discussion

### 3.1. Aluminium and Sulphur Concentrations

**Figure 1:**
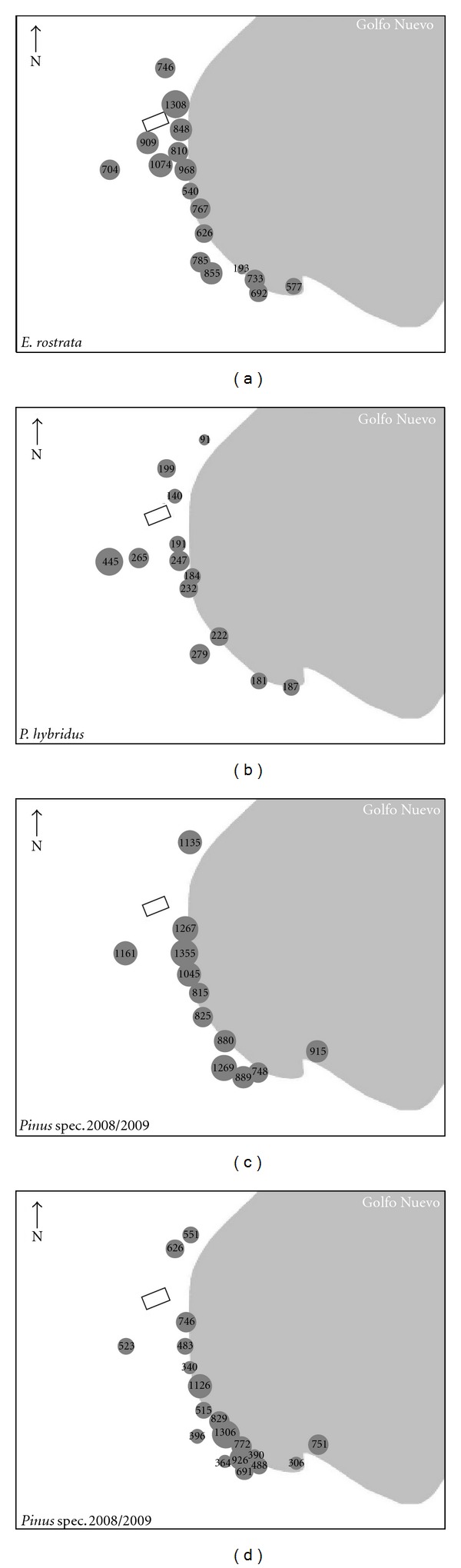
Aluminium concentration (*μ*g g^−1^ DW) in the study area determined in leaves of *E. rostrata* (a) and *P. hybridus* (b) and needles of *Pinus* spec. 2008/2009 (c) and 2009/2010 (d).

The Al concentrations ranged between 89.49 and 1354 *μ*g g^−1^ DW in the deciduous tree species, while they varied between 284.2 and 1442 *μ*g g^−1^ DW in the conifers (Table 1; Figure 1). Among the deciduous species, *E. rostrata* showed the highest accumulation of Al per leaf area (an average of 11.43 *μ*g cm^−2^) in comparison with *P. hybridus* (average of 0.718 *μ*g cm^−2^). Area of *Pinus* needles could not be determined so that these area-related concentrations are missing for the conifer. However, the comparison between the Al concentrations in *Pinus* spec. needles from different ages showed higher values in the older needles than in the current year needles, reflecting a greater accumulation of aluminium with a longer exposure time (Table 1). Pearson's correlation coefficients for the relationship between Al and F concentrations were *r* = 0.4 (*P* = 0.106) for *E. rostrata*, *r* = 0.01 (*P* = 0.975) for *P. hybridus*, *r* = 0.582 (*P* = 0.047) for needles of *P. spec.* from the year 2009, and *r* = 0.292 (*P* = 0.225) for pine needles stemming from the year 2010. Although the accumulation of Al in plants is dependent on the species, these concentrations are similar compared to results of other studies employing different species in highly contaminated areas; for example, in Spain some authors have reported Al concentrations between 852 and 4593 ppm in plants of the genus *Eucalyptus* and between 127 and 1732 for the genus *Pinus* [29, 30]; for *Populus* Laureysens et al. [31] found concentrations between 100 and 250 ppm in plants growing in Belgium.

**Figure 2:**
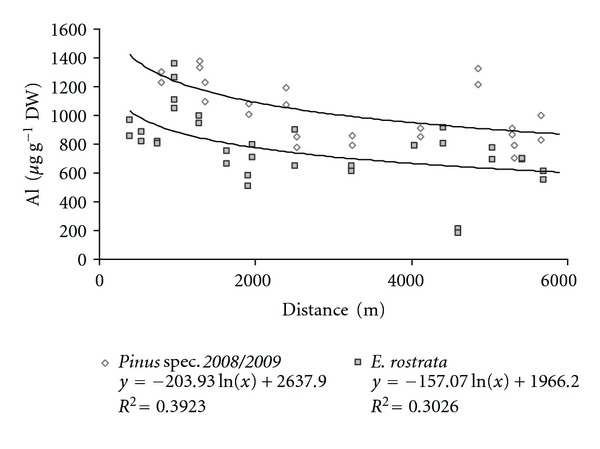
Aluminium pollution gradient in the study area expressed as Al concentration in *Pinus* spec. one-year-old needles (dashed lines) and *E. rostrata* (full lines) in relation to the distance from the emission source.

However, it should be noted that there are some species, mainly conifers, that tend to acidify soils and therefore are associated with degradation processes releasing Al ions from the bedrock [29, 32–34]. Therefore, the presence of acidifying species in Al-polluted soil may generate a potential risk situation not only for these plant species but also for other species growing in the area. On the other hand, numerous studies report on the negative effect of high levels of soluble Al on plant growth [29, 35, 36]. Furthermore, high concentrations of Al in the food and drinking water generate a potentially hazardous situation for the human health considering that it has recently been established that Alzheimer's disease is associated with the Al content in the human brain [11]. In addition, the Al concentrations obtained in this study indicate a pollution gradient between the distance to the aluminium smelting and Al concentrations in the leaves of *E. rostrata* and needles of *Pinus* spec. from the last years (Figure 2).

**Figure 3:**
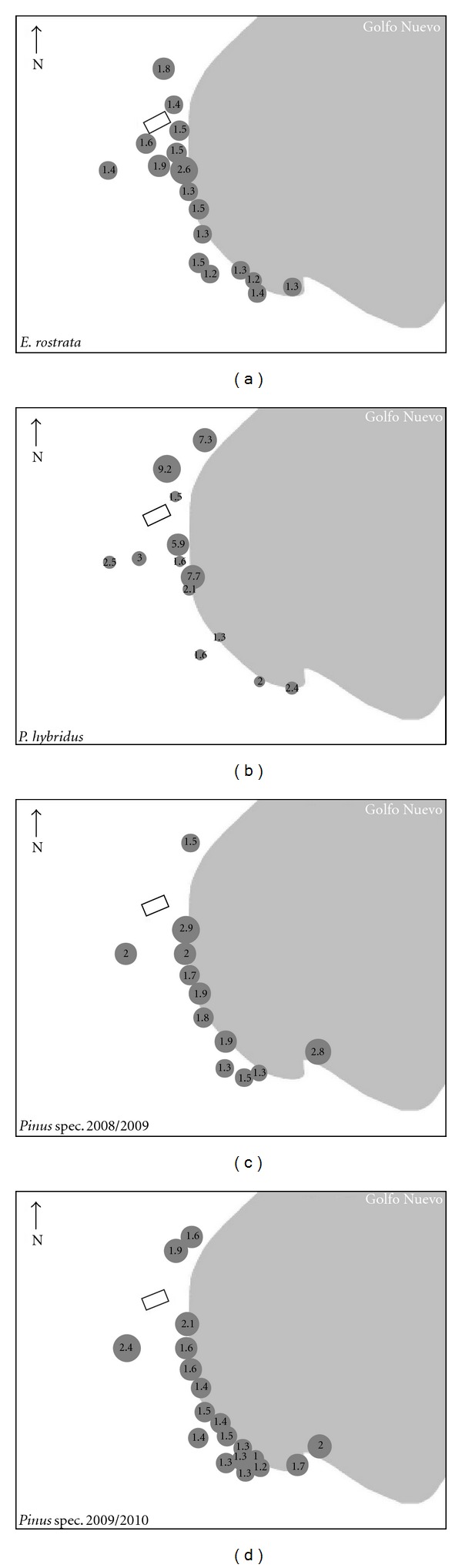
Sulphur concentration (mg g^−1^) in the study area determined in leaves of *E. rostrata* (a) and *P. hybridus* (b) and needles of *Pinus* spec. 2008/2009 (c) and 2009/2010 (d).

Sulphur concentrations were found to range between 1.153 and 9.343 mg g^−1^ DW in the deciduous tree species and between 0.985 and 3.046 in the conifers (Table 1; Figure 3). Although all species studied indicate high sulphur accumulation, leaves of *P. hybridus* showed the greatest accumulation in relation to other species, which could be related to specific leaf morphological characteristics. A study of foliar sulphur concentrations in four tree species of *Ficus* nearby to urban and industrial sources in China showed values from 3.3 × 10^3^ to 5.3 × 10^3^ mg kg^−1^ DW [37]. Therefore, the concentrations found in this study are among those found in industrial zones. In addition, the S concentrations in *E. rostrata *(*R* ^2^ = 0.21) and *Pinus* spec. (2008-2009; (*R* ^2^ = 0.25)) showed only a very slight inverse relationship to the distance to the industry. It can thus be followed that sulphur concentrations are also affected by the emissions from vehicular traffic and other anthropogenic sources.

### 3.2. Physiological Parameters

Table 1 shows the mean, standard deviation, minimum, and maximum values of the physiological parameters measured in poplar, *Eucalyptus*, and pine. Total chlorophyll contents were similar between deciduous tree species and were greater than those in conifers. Chlorophyll degradation parameters measured (Chl-b/Chl-a and Phe-a/Chl-a) showed the highest mean values in the needles of *Pinus* spec. for the previous year. Regarding membrane lipid oxidation products (HPCD and MDA), the species *P. hybridus* and *Pinus* spec. (2008-2009) showed higher mean values, results that are consistent with the highest mean aluminium and sulphur values. These findings indicate a relationship between emissions of these compounds and membrane lipid oxidation products in foliage.

Significant results were found for correlations between aluminium, sulphur, and physiological parameters in the foliage of the tree species analyzed. In *E. rostrata* a positive correlation was found between concentrations of aluminium and sulphur (*r* = 0.38), MDA, Phe-a/Chl-a, and HPCD (*r* = 0.40; *r* = 0.68). In *Pinus* needles from the previous year a positive correlation was found between Al and MDA and with total chlorophyll (*r* = 0.39; *r* = 0.37), HPCD and MDA (*r* = 0.44). Finally, *Pinus* needles from the current year showed a negative correlation between MDA and total chlorophylls and Phe-a/Chl-a (*r* = −0.41; *r* = −0.48) and a positive correlation between MDA and HPCD (*r* = 0.68).

## 4. Conclusion

The analysis of Al and sulphur concentrations in leaves and needles confirmed a significant pollution gradient with the highest values in samples collected in the vicinity of the emission source. The species *E. rostrata* and *Pinus* spec. (2008-2009) showed a steep pollution gradient for Al and a slight gradient for sulphur. However, the pollution gradient for fluoride was steeper, indicating that the latter may be deposited as a gas, while Al is associated to particle deposition. Regarding the impact of the aluminium smelter, the results of this study show that it is severe in an area close to the source, and considering the possibility of contaminated soil acidification especially by aluminium, further studies in relation to the environmental and human health should be carried out.

## Figures and Tables

**Table 1:** Mean, standard deviation, minimum, and maximum values of aluminium, sulphur, chlorophyll a+b concentration, Chl-b/Chl-a, Ph-a/Chl-a, HPCD, and MDA measured in foliage of different tree species in Puerto Madryn, Argentina.

Species	Descriptive statistics	Aluminium (*μ*g g^−1^ DW)	Sulphur (mg g^−1^ DW)	Chl-a+b (mg g^−1^ DW)	Chl-b/Chl-a	Phe-a/Chl-a	HPCD (*μ*mol g^−1^ DW)	MDA (nmol g^−1^ DW)
*P. hybridus* *N* = 13	Mean	220.2	3.706	2.19	0.33	1.44	51.8	290
	SD	84.48	2.717	0.88	0.09	0.17	28.4	95.2
	Min	89.49	1.290	0.89	0.13	1.04	20.5	141
	Max	467.0	9.343	4.65	0.48	1.76	124	468

*E. rostrata* *N* = 17	Mean	772.6	1.508	2.07	0.42	1.35	109	224
	SD	239.6	0.380	0.76	0.27	0.21	33.2	89.1
	Min	181.3	1.153	0.73	0.04	1.05	55.9	120
	Max	1354.3	2.788	4.34	1.52	1.95	186	446

*Pinus *spec. (2008/2009) *N* = 13	Mean	1025	1.887	1.43	0.46	2.09	147	256
	SD	208.1	0.514	0.50	0.13	0.59	52.3	97.7
	Min	704.4	1.241	0.76	0.31	1.41	57.9	163
	Max	1378	3.046	2.88	0.75	3.29	244	547

*Pinus *spec. (2009/2010) *N* = 19	Mean	638.3	1.555	1.31	0.44	1.47	141	279
	SD	271.9	0.356	0.65	0.13	0.24	52.1	60.8
	Min	284.2	0.985	0.53	0.23	0.82	27.6	174
	Max	1442	2.450	2.90	0.70	2.03	285	449

## References

[1] Aboal JR, Couto JA, Fernández JA, Carballeira A (2008). Physiological responses to atmospheric fluorine pollution in transplants of Pseudoscleropodium purum. *Environmental Pollution*.

[2] Real C, Aboal JR, Fernández JA, Carballeira A (2003). The use of native mosses to monitor fluorine levels—and associated temporal variations—in the vicinity of an aluminium smelter. *Atmospheric Environment*.

[3] Vike E, Håbjørg A (1995). Variation in fluoride content and leaf injury on plants associated with three aluminium smelters in Norway. *Science of the Total Environment*.

[4] Vike E (1999). Air-pollutant dispersal patterns and vegetation damage in the vicinity of three aluminium smelters in Norway. *Science of the Total Environment*.

[5] Knutzen J (1995). Effects on marine organisms from polycyclic aromatic hydrocarbons (PAH) and other constituents of waste water from aluminium smelters with examples from Norway. *Science of the Total Environment*.

[6] Kuo SC, Hsieh LY, Tsai CH, Tsai YI (2007). Characterization of PM2.5 fugitive metal in the workplaces and the surrounding environment of a secondary aluminum smelter. *Atmospheric Environment*.

[7] Vike E (2005). Uptake, deposition and wash off of fluoride and aluminium in plant foliage in the vicinity of an aluminium smelter in Norway. *Water, Air, and Soil Pollution*.

[8] Rey-Asensio A, Carballeira A (2007). Lolium perenne as a biomonitor of atmospheric levels of fluoride. *Environment International*.

[9] Klumpp A, Klumpp G, Ansel. W, Klumpp A, Ansel W, Klumpp G (2004). Urban air quality in Europe- results of three years of standardized biomonitoring studies. *Urban Air Pollution, Bioindication and Environmental Awareness*.

[10] James PS, Raveendra VI, Timothy EF (1995). Multivariate statistical examination of spatial and temporal patterns of heavy metal contamination in New Bedford Harbor marine sediments. *Environmental Science and Technology*.

[11] McLachlan DRC (1995). Aluminium and the risk for Alzheimer’s disease. *Environmetrics*.

[12] Müller M, Anke M, Illing-Günther H (1998). Aluminium in foodstuffs. *Food Chemistry*.

[13] Wong MH, Zhang ZQ, Wong JWC, Lan CY (1998). Trace metal contents (Al, Cu and Zn) of tea: tea and soil from two tea plantations, and tea products from different provinces of China. *Environmental Geochemistry and Health*.

[14] Klumpp A, Klumpp G, Domingos M (1996). Bio-indication of air pollution in the tropics: the active monitoring programme near Cubatão (Brazil). *Gefahrstoffe Reinhaltung der Luft*.

[15] Zaballa Romero M, Stabentheiner E, Kosmus W, Gössler W, Lazar R, Grill D (2005). The use of bioindication plants for the assessment of air pollutants in the city of Cochabamba, Bolivia. *Phyton*.

[16] Ares JO (1978). Fluoride cycling near a coastal emission source. *Journal of the Air Pollution Control Association*.

[17] Ares JO, Villa A, Mondadori G (1980). Air pollutant uptake by xerophytic vegetation: fluoride. *Environmental and Experimental Botany*.

[18] Ares JO, Villa A, Gayoso AM (1983). Chemical and biological indicators of fluoride input in the marine environment near an industrial source (Argentina). *Archives of Environmental Contamination and Toxicology*.

[19] Murphy CE, Ares J (1982). The uptake of hydrogen fluoride by a forest. *Ecological Modelling*.

[20] Comisión Nacional de Valores http://www.cnv.gov.ar/InfoFinan/Zips.asp?Lang=0&CodiSoc=7&DescriSoc=Aluar%20Aluminio%20Argentino&Letra=A&TipoDocum=1&TipoArchivo=1&TipoBalance=2.

[21] Moodys http://www.moodys.com.ar/PDF/Empresas/28-12-12/Aluar_Revision%20triml_Sep%2011.pdf.

[22] Rodriguez JH, Wannaz ED, Pignata ML, Fangmeier A, Franzaring J Fluoride biomonitoring around a large aluminium smelter in Argentina using foliage from different tree species.

[23] INDEC http://www.indec.gov.ar/webcenso/index.asp.

[24] CENPAT http://www.cenpat.edu.ar.

[25] VDI (2007). *VDI 3957 Part 11: Biological Measuring Techniques for the Determination and Evaluation of Effects of Air Pollution on Plants (bioindication)—Sampling of Leaves and Needles for a Biomonitoring of the Accumulation of Air Pollutants (passive biomonitoring), VDI/DIN-Handbuch Reinhaltung der Luft—Band 1A: Maximale Immissions-Werte; Band 1B: Umweltmeteorologie, Kommission Reinhaltung der Luft im VDI und DIN—Normenausschuss KRdL*.

[26] Carreras HA, Gudiño GL, Pignata ML (1998). Comparative biomonitoring of atmospheric quality in five zones of Cordoba city (Argentina) employing the transplanted lichen Usnea sp. *Environmental Pollution*.

[27] Wannaz ED, Pignata ML (2006). Calibration of four species of Tillandsia as air pollution biomonitors. *Journal of Atmospheric Chemistry*.

[28] González CM, Pignata ML (1994). The influence of air pollution an soluble proteins, chlorophyll degradation, MDA, sulphur and amounts of heavy metals in a transplanted lichen. *Chemistry and Ecology*.

[29] Álvarez E, Fernández-Marcos ML, Monterroso C, Fernández-Sanjurjo MJ (2005). Application of aluminium toxicity indices to soils under various forest species. *Forest Ecology and Management*.

[30] Rossini Oliva S, Mingorance MD (2006). Assessment of airborne heavy metal pollution by aboveground plant parts. *Chemosphere*.

[31] Laureysens I, Blust R, De Temmerman L, Lemmens C, Ceulemans R (2004). Clonal variation in heavy metal accumulation and biomass production in a poplar coppice culture: I. Seasonal variation in leaf, wood and bark concentrations. *Environmental Pollution*.

[32] Álvarez E, Monterroso C, Fernández Marcos ML (2002). Aluminium fractionation in Galician (NW Spain) forest soils as related to vegetation and parent material. *Forest Ecology and Management*.

[33] Augusto L, Bonnaud P, Ranger J (1998). Impact of tree species on forest soil acidification. *Forest Ecology and Management*.

[34] Messenger AS (1980). Spruce plantation effects on soil moisture and chemical element distribution. *Forest Ecology and Management*.

[35] Adams F, Hathcock PJ (1984). Aluminum toxicity and calcium deficiency in acid subsoil horizons of two Coastal Plains soil series. *Soil Science Society of America Journal*.

[36] Mohapatra S, Cherry S, Minocha R (2010). The response of high and low polyamine-producing cell lines to aluminum and calcium stress. *Plant Physiology and Biochemistry*.

[37] Qiu Y, Guan D, Song W, Huang K (2009). Capture of heavy metals and sulfur by foliar dust in urban Huizhou, Guangdong Province, China. *Chemosphere*.

